# Validation of an optimised protocol for quantification of microplastics in heterogenous samples: A case study using green turtle chyme

**DOI:** 10.1016/j.mex.2018.07.009

**Published:** 2018-08-10

**Authors:** Alexandra G.M. Caron, Colette R. Thomas, Kathryn L.E. Berry, Cherie A. Motti, Ellen Ariel, Jon E. Brodie

**Affiliations:** a Australian Institute of Marine Science PM3, Townsville MC, QLD 4810, Australia; b Centre for Tropical Water and Aquatic Ecosystem Research (TropWATER), James Cook University, Townsville 4811, Australia; c AIMS@JCU, Australian Institute of Marine Science and James Cook University, Townsville, Australia; d SEED Science, Australia; e College of Public Health, Medical and Veterinary Sciences, James Cook University, Townsville 4811, Australia; f ARC Centre of Excellence for Coral Reef Studies, James Cook University, Townsville 4811, Australia

**Keywords:** Sequential protocol for extracting microplastics from heterogenous sample matrices, SLS, sodium lauryl sulphate, PE, polyethylene, HDPE, high density polyethylene, PVC, polyvinyl chloride, PP, polypropylene, PET, poly(ethylene terephthalate), AM-PS, (aminoethyl) polystyrene, RO, reverse osmosis, ATR-FTIR, attenuated total reflectance Fourier transform-infrared spectroscopy, Plastic ingestion, Marine debris, Plastic contamination, Extraction technique, Chemical digestion, Density separation, Fourier transform-infrared spectroscopy

## Abstract

Quantifying the extent of microplastic (<5 mm) contamination in the marine environment is an emerging field of study. Reliable extraction of microplastics from the gastro-intestinal content of marine organisms is crucial to evaluate microplastic contamination in marine fauna. Extraction protocols and variations thereof have been reported, however, these have mostly focussed on relatively homogenous samples (i.e. water, sediment, etc.). Here, we present a microplastic extraction protocol for examining green turtle (*Chelonia mydas*) chyme (i.e. ingested material and digestive tract fluid), which is a heterogeneous composite of various organic dietary items (e.g. seagrass, jellyfish) and incidentally-ingested inorganic materials (sediment). Established extraction methods were modified and combined. This protocol consists of acid digestion of organic matter, emulsification of residual fat, density separation from sediment, and chemical identification by Fourier transform-infrared spectroscopy. This protocol enables the extraction of the most common microplastic contaminants>100 μm: polyethylene, high-density polyethylene, (aminoethyl) polystyrene, polypropylene, and polyvinyl chloride, with 100% efficiency. This validated protocol will enable researchers worldwide to quantify microplastic contamination in turtles in a reliable and comparable way.

• Optimization of microplastic extraction from multifarious tissues by applying established methods in a sequential manner.
• Effective for heterogenous samples comprising organic and inorganic material.

## Graphical abstract

**Figure:**
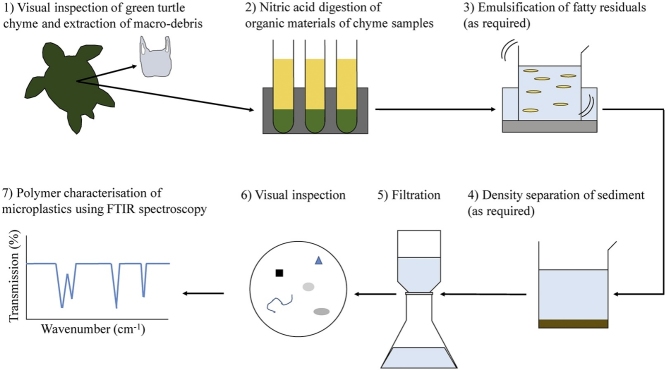

**Specifications Table**

**Table:** 

Subject area	*Environmental Science*
More specific subject area	*Marine pollution*
Method name	*Sequential protocol for extracting microplastics from heterogenous sample matrices*
Name and reference of original method	*Nitric acid digestion:* Claessens, M., Van Cauwenberghe, L., Vandegehuchte, M.B., Janssen, C.R., 2013. New techniques for the detection of microplastics in sediments and field collected organisms. Mar. Pollut. Bull. 70, 227–233. doi:https://doi.org/10.1016/j.marpolbul.2013.03.009 *Density separation:* Hidalgo-Ruz, V., Gutow, L., Thompson, R.C., Thiel, M., 2012. Microplastics in the marine environment: A review of the methods used for identification and quantification. Environ. Sci. Technol. 46, 3060–3075. doi:https://doi.org/10.1021/es2031505 *FTIR spectroscopy:* Hidalgo-Ruz, V., Gutow, L., Thompson, R.C., Thiel, M., 2012. Microplastics in the marine environment: A review of the methods used for identification and quantification. Environ. Sci. Technol. 46, 3060–3075. doi:https://doi.org/10.1021/es2031505 Kroon, F., Motti, C., Talbot, S., Sobral, P., Puotinen, M., 2018. A workflow for improving estimates of microplastic contamination in marine waters: A case study from North-Western Australia. Environmental Pollution, 238, 26–38. Thompson, R.C., Olsen, Y., Mitchell, R.P., Davis, A., Rowland, S.J., John, A.W.G., McGonigle, D., Russell, A.E., 2004. Lost at sea: Where is all the plastic? Science 304, 838. doi:https://doi.org/10.1126/science.1094559 Jung, M.R., Horgen, F.D., Orski, S. V, C, V.R., Beers, K.L., Balazs, G.H., Jones, T.T., Work, T.M., Brignac, K.C., Royer, S., Hyrenbach, K.D., Jensen, B.A., Lynch, J.M., 2018. Validation of ATR FT-IR to identify polymers of plastic marine debris, including those ingested by marine organisms. Mar. Pollut. Bull. 127, 704–716. doi:https://doi.org/10.1016/j.marpolbul.2017.12.061
Resource availability	Hardware: Extraction: • Aluminium trays and foil • Block heater (80 °C, model AIM500, SEAL Analytical) • Branson^®^ 2200 Ultrasonic Clearer • Covered glass dishes (shallow, flat bottomed, 5 cm diameter) • Freezer (−20 °C) • Fridge (4 °C) • Glass beakers (200 mL) • Glass Pasteur pipette • Glass stirring rod • Glass test tubes (50 mL) • Glass Vacuum-filter apparatus (glass frit Buchner funnel with metal clamp and vacuum Erlenmeyer flask; Millipore) • Hot plate (50 °C, Stuart CB160) • Lint free tissue • Maggylamp (×2 magnification) • Metal bucket • Metal needles • Metal tweezers • Metal teaspoon • Millipore HA cellulose nitrate/acetate 0.45 μm pore membrane filters • Oven (60 °C) • Paper clip • Pliable steel fabric with a mesh size of 100 μm • Steel mesh sieve (100 μm, 20 cm diameter) Analysis: • PerkinElmer Spectrum 100 Infrared Spectrometer with Attenuated Total Reflectance Software: • PerkinElmer Spectrum (V. 10.5.4); NICODOM IR libraries (Polymers and Additives, Coatings, Fibres, Dyes and Pigments, Petrochemicals Full Version; NICODOM Ltd., Czech Republic) Reagents: • Chemical digestion: Nitric acid (HNO_3_, 69.5%, Scharlau) • Density separation: NaCl solution, 1.2 g/cm^3^, Sigma Aldrich • Emulsification: sodium lauryl sulphate pellets (SLS, Acros Organics, CAS number 151-21-3) • Fourier transform-infrared spectroscopy: methanol • Reverse osmosis water

## Method details

### Protocol background

Plastics are ubiquitous and widespread in the marine environment; polluting the ocean surface, water column, and benthos [[1], [2], [3]]. They are broadly divided into two categories; macroplastics (>5 mm) and microplastics (<5 mm, [4,5]), and mainly impact marine life through the processes of entanglement and ingestion [6]. All seven turtle species are known to be affected by plastic debris globally [[7], [8], [9], [10]]. Unlike other marine organisms, the herbivorous feeding strategies of green turtles make them highly susceptible to ingestion of marine debris [11,12]. Plastic debris can become entangled among green turtle food sources such as seagrass leaves and macroalgae [13]. The backward-facing oesophageal papillae of turtles inhibits regurgitation and facilitates particle accumulation in the gut [[14], [15], [16]]. Chyme (i.e. ingested material and digestive tract fluid) from non-pelagic green turtles is expected to be relatively complex, comprising a range of organic (plant and animal material) and inorganic (mineral and sediment) matrices, as their broad diet includes jellyfish, sponges, seagrass, algae and associated sediments and epibionts [37]. Therefore, a protocol capable of efficiently extracting microplastics from all matrices is required to accurately establish contamination levels.

Methods for extracting microplastics have been developed for a range of relatively homogeneous sample matrices and include: visual assessment using microscopy [17], density separation using hypersaline solutions [[18], [19], [20], [21]] and chemical digestion [18,[22], [23], [24]]. Many of these methods are suitable and efficient for either homogenously organic or homogenously inorganic sample matrices; however, each of them alone are not suitable for microplastic extraction from green turtle chyme. Here we test several microplastic extraction methods and reagent variations in combination using a process of elimination to identify the optimal steps necessary to establish a robust and reliable way to extract and quantify microplastic contamination in a variety of heterogeneous marine organism tissues.

### Material and methods

**Fig. 1:**
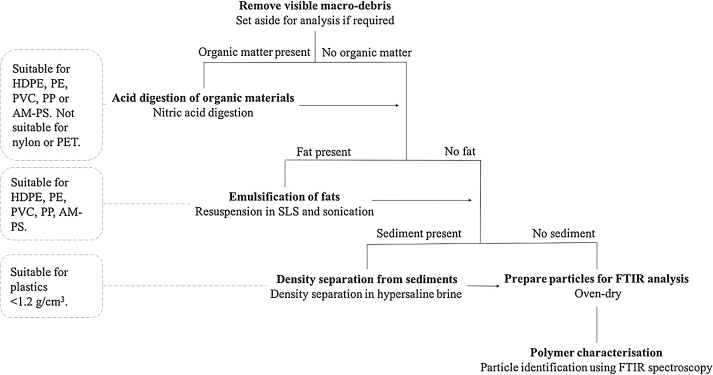
Sequential extraction protocol for the recovery of the most prevalent microplastic pollutants from green turtle chyme showing polymer suitability. List of abbreviations: sodium lauryl sulphate (SLS), polyethylene (PE), high density polyethylene (HDPE), polyvinyl chloride (PVC), polypropylene (PP), poly(ethylene terephthalate) (PET), (aminoethyl) polystyrene (AM-PS).

A summary of the microplastic sequential extraction protocol is presented in Fig. 1.

To prevent procedural contamination, all lab work surfaces and equipment should be rinsed with reverse osmosis (RO) water, work must be conducted in a fume hood and plastic tools and containers should be avoided.

#### Sample preparation

##### Materials

• Aluminium trays and foil
• Freezer (−20 °C)
• Fridge (4 °C)
• Large glass beaker or metal bucket, to fit chyme
• Large metal spoon or ladle
• RO water
• Steel mesh sieve (100 μm, 20 cm diameter)

##### Procedure

• Green turtles that had been either found dead or required euthanizing were kindly made available by Queensland Parks and Wildlife Service (QPWS). Necropsies were undertaken by a qualified veterinary pathologist. Foreguts (including oesophagus, stomach, and small intestine) were necropsied, the rest of the digestive tract being required for a different study. Anterior and posterior extremities of the tracts were closed with non-plastic strings prior to removal. Tracts were extracted from turtle cavities and stored in aluminium trays and foil, and frozen at −20 °C within a few hours. Tracts were thawed in a fridge at 4 °C for 48 h prior to analysis.
• Collect chyme from green turtle digestive tract and transfer it into a glass or metal container. Most of the chyme can be collected using a metal spoon or ladle. To collect chyme adhered to the gastro-intestinal tract, rinse tract with RO water over a 100 μm steel mesh sieve and add retained residue to the chyme. The filtrate, being <100 μm is discarded.
• Visually inspect the chyme and remove any visible macro-debris (i.e. rope, fishing line, bags, bottle lids, etc.). Rinse them with RO water over 100 μm steel mesh sieve and add retained residue to the chyme to assure minimal loss of potential microplastics. The filtrate, being <100 μm is discarded.
• Homogenise the chyme by manual stirring for one minute with a metal spoon (excessive mechanical stirring or sonication should be avoided to reduce the likelihood of larger brittle plastic items further fragmenting). Thorough homogenisation of the chyme is mandatory especially if analysing only a subsample of the whole chyme, as plastic debris tend to accumulate in some parts of green turtle digestive tract [7,25].
• Weigh the chyme sample to determine the number of test tubes and volume of HNO_3_ required (see *Acid digestion of organic materials).*

#### Acid digestion of organic materials

##### Materials

• Aluminium foil
• Block heater (80 °C, model AIM500, SEAL Analytical)
• Glass beaker (500 mL, or to fit volume of samples and HNO_3_)
• Glass stirring rod
• Glass test tubes (50 mL)
• Metal teaspoon
• Nitric acid (HNO_3_, 69.5%, Scharlau)
• Paper clip
• Pliable steel fabric with a mesh size of 100 μm
• RO water

##### Procedure

• Put ∼6 g wet weight (w/w) of homogenised chyme per 50 mL test tube using a metal teaspoon. Where chyme from an individual is >6 g, the sample should be split and multiple test tubes used.
• Add 18 mL of HNO_3_ to each sample (ratio 3:1, HNO_3_ mL: chyme g w/w).
• Place the test tubes into the block heater. Cover the test tubes with aluminium foil and let them sit overnight at room temperature (∼20 °C).
• Uncover the test tubes and heat samples for two hours at 80 °C in the block heater. Test tubes should be closely monitored. They can be cautiously stirred with a glass stirring rod to make sure all materials remain immersed and to minimise foaming.
• Filter samples while still warm over the 100 μm steel mesh sieve. To filter, fold the 100 μm mesh into a conical shape and secure with a paper clip to a suitably-sized glass beaker. Allow retained material to gather at the bottom of the cone for ease of rinsing and collection.
• Discard the acid solution down the sink with ten times its volume of tap water to neutralize it.
• Rinse collected materials with RO water over the sink and discard filtrate. While still in the sieve, visually inspect the digested sample for undigested fat residue and sediment. If fat residues are observed proceed with the emulsification method (Fig. 1; see *Emulsification of fats*). If no fat residues are observed but sediment is present, proceed with the density separation method (see *Density separation from sediments*). If no fat residues or sediment are present, proceed with filtration (see *Filtration*).

##### Note

Chyme from different turtles can have different reactivity to HNO_3_, especially during the heating step. Very reactive samples can produce an unmanageable amount of hazardous foaming. Running a test prior to applying this method to a large number of samples is recommended. Reduce the quantity of sample in the test tube if the chyme reacts too strongly to HNO_3_.

Depending on the composition and complexity of the samples, multiple samples from one individual can be combined at the filtration step. Act according to the amount of residue retained. Combining the residue reduces the number of samples to be processed in subsequent steps and streamlines the overall process.

#### Emulsification of fats

**Fig. 2:**
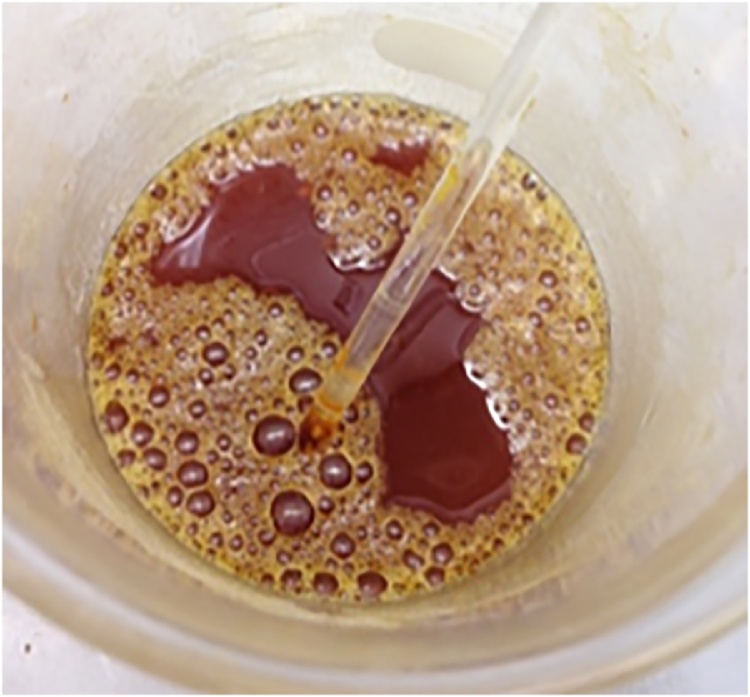
Fat residue remaining after nitric acid digestion of green turtle chyme during a preliminary test of the protocol.

Proceed with an emulsification if an intractable fat residue remains after acid digestion of samples (Fig. 2).

##### Materials

• Glass beaker (200 mL)
• Glass Pasteur pipette
• Glass stirring rod
• Hot plate (50 °C, Stuart CB160)
• Metal teaspoon
• Paper clip
• Pliable steel fabric with a mesh size of 100 μm
• RO water
• Sodium lauryl sulphate pellets (SLS, 100%, Acros Organics, CAS number 151-21-3)
• Ultrasonic bath (47 kHz ± 6%, Branson^®^ 2200 Ultrasonic Clearer)

##### Procedure

• Prepare a warm 1 g/L SLS solution by adding SLS pellets to RO water on a 50 °C hot plate. Use the glass stirring rod to stir until well dissolved and keep at 50 °C on the hot plate.
• Re-suspend all retained residue (i.e. collected on the 100 μm sieve, see *Acid digestion of organic materials*) remaining from the acid digestion in the warm SLS solution. To do so, place the sieve mesh upside-down over a 200 mL glass beaker and rinse it using a glass pipette and the warm SLS solution (∼50 mL or enough to remove all residue). Once all materials are transferred into the glass beaker, add extra SLS solution to give a final volume of 200 mL if required.
• Sonicate for 30 s in an ultrasonic bath (47 kHz ± 6%). Avoid extended sonication to reduce the likelihood of larger brittle plastic items further fragmenting.
• Filter samples while still warm over the 100 μm sieve (as done in *Acid digestion of organic materials*; fold the 100 μm mesh into a conical shape and secure with a paper clip to a suitably-sized glass beaker).
• Discard SLS solution down the sink with ten times its volume of tap water to neutralize it.
• Repeat the operation until no fat residue is observed on the sieve.
• Rinse retained materials with RO water over the sink and discard filtrate. While still in the sieve, visually inspect retained materials for sediment. If sediment is observed, proceed with the density separation method (Fig. 1, see *Density separation from sediments*). If no sediment is observed, proceed with filtration (see *Filtration*).

##### Note

This emulsification method was successful in dissolving droplets of fats. If the amount of fat present in the samples is large, such as chunks, or forms a thick layer, further homogenization may be necessary, or different digestions could be tested. This could include sample digestion using KOH (needing from a few days to a few weeks processing) which has been successfully applied to stomach content of fish [22,26]. If combining samples (from the same individual) after acid digestion, make sure to visually inspect the retained materials on the sieve; it is important to maintain a manageable amount of fat for the emulsification method.

#### Density separation from sediments

**Fig. 3:**
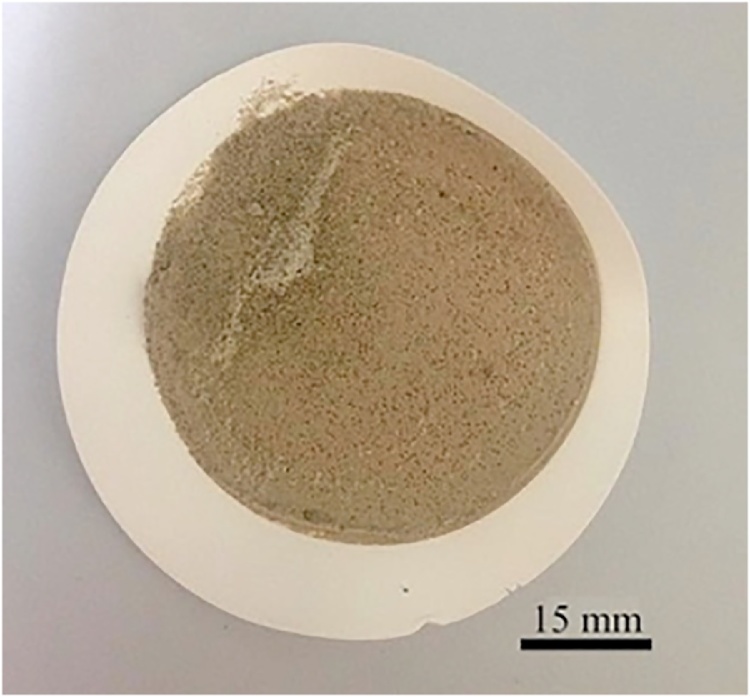
Sediments present after acid digestion of turtle chyme during a preliminary test of the protocol.

Proceed with density separation if sediment is present after filtration (i.e. either after acid digestion or after emulsification; Fig. 3).

##### Materials

• Aluminium foil
• Glass beakers (200 mL)
• Glass Pasteur pipette
• Glass stirring rod
• NaCl (Sigma Aldrich)
• RO water

##### Procedure

• Prepare a hypersaline brine (NaCl, 1.2 g/cm^3^) by saturating RO water with NaCl (water solubility at 20 °C = 357 g/L). NaCl must be added while continuously stirring until it no longer dissolves.
• Re-suspend all retained materials from the previous method used (either emulsification or acid digestion) in 200 mL of the hypersaline brine. To do so, place the sieve mesh upside-down over a 200 mL glass beaker and rinse it using a glass pipette and the hypersaline brine (∼50 mL or enough to remove all retained materials). Once transferred, add extra hypersaline brine to give a final volume of 200 mL.
• Stir the solution with the glass stirring rod for 30 s. Rinse the stirring rod with the brine into the beaker containing the sample.
• Cover the beaker with aluminium foil and allow to settle for 1 h.
• Collect the top ∼20 mL of the solution using a glass Pasteur pipette and transfer it to a clean 200 mL glass beaker.
• Rinse the pipette three times with RO water into the clean 200 mL glass beaker to remove any particles that may have adhered to the internal or external walls of the pipette. Discard the remaining ∼180 mL of brine and sediment.

##### Note

Make sure NaCl is free of plastic contamination; where possible use scientific grade NaCl. NaCl in soft plastic bags or in plastic mesh bags can become contaminated with microplastics as bags age and degrade. In addition, ensure the salt is stored in a covered, clean container, to avoid microfiber contamination while the salt is exposed to the lab air. Regardless, prepared NaCl solutions should be checked for contamination prior to undertaking density separation. If necessary, the solution should be filtered through cellulose nitrate/acetate 0.45 μm pore membrane filters (Millipore HA).

#### Filtration

##### Materials

• Covered glass dishes (shallow, flat bottomed, 5 cm diameter). Adapted size to match the filters.
• Glass Pasteur pipette
• Glass Vacuum-filter apparatus (glass frit Buchner funnel with metal clamp and vacuum Erlenmeyer flask; Millipore)
• Metal tweezers
• Millipore HA cellulose nitrate/acetate 0.45 μm pore membrane filters, larger (>100 μm) pore sizes can be used to selectively recover microplastics in different size ranges
• Oven (60 °C)
• RO water

##### Procedure

• Filter the collected (low density) supernatant from the density separation method under vacuum onto 0.45 μm pore membrane filters. With RO water, rinse the wall of the 200 mL glass beaker into the vacuum-filter apparatus a minimum of three times (or as much as needed) to recover all particles. If the density separation method was not used (i.e. no sediment was present in the samples), materials collected on the 100 μm sieve after HNO_3_ digestion or after emulsification should be vacuum-filtered as above.
• Rinse the wall of the vacuum-filter funnel three times with RO water using a pipette to ensure all particles are on the filter.
• Carefully place each filter in a covered glass dish using metal tweezers.
• Oven-dry at 60 °C for 4 h.
• Visually inspect filters for microplastics [17]. Characterise particles using FTIR spectroscopy (see *Polymer characterisation*).

#### Polymer characterisation

Polymer composition of recovered microplastics can be determined using FTIR spectroscopy [21,22,[27], [28], [29], [30]].

##### Instrument and software

• PerkinElmer Spectrum 100 infrared spectrometer with attenuated total reflectance (ATR-FTIR) operated with PerkinElmer Spectrum (V. 10.5.4) software
• NICODOM IR libraries (Polymers and Additives, Coatings, Fibres, Dyes and Pigments, Petrochemicals Full Version; NICODOM Ltd., Czech Republic)

##### Materials

• Lint free tissue (for cleaning)
• Maggylamp (×2 magnification)
• Metal tweezers and needles
• Methanol (for cleaning)

##### Procedure

• No additional treatment of the micro-particles is necessary prior to FTIR analysis. Macroplastics recovered during the visual inspection of the chyme (see *Sample preparation*) should be rinsed thoroughly with RO water and oven-dried at 60 °C for 4 h before analysis by ATR-FTIR.
• Place individual items on the ATR diamond using tweezers or needles. Use a pressure clamp to ensure good contact with the sample.
• Acquire FTIR spectra in transmission mode on a PerkinElmer Spectrum 100 FTIR Spectrometer using an attenuated total reflectance (ATR) accessory as per Kroon et al. [29]. Use the Data Tune-up command to smooth and perform a baseline correction for all spectra using default PerkinElmer parameters. In this study spectral data were accumulated for 64 scans at 4 cm^−1^ resolution with a wavenumber range of 4000–400 cm^−1^ [22,27] using Euclidian distance against commercially available NICODOM IR libraries and a percent match between the reference spectra and the sample obtained.
• As per Kroon et al. [29] samples with a percent match of <60% were considered a low match, 60–<70% an intermediate match and ≥70–100% a high match. All spectra must be further inspected and any unexplained bands investigated by reviewing the lower percent matches and the literature to confirm polymer identification.

### Protocol validation and suitability

#### Protocol validation

The extraction efficiency of the sequential protocol was assessed by spiking three 6 g (w/w) homogenised chyme samples (see *Sample preparation*) with five micro-beads (high-density polyethylene (HDPE); 192 ± 48 μm). Spiked samples were then processed with the sequential protocol. Since both fat and sediments were present in these samples, all three extraction methods: acid digestion, emulsification of fats, and density separation, were used. Every micro-bead was recovered; the extraction efficiency of the extraction protocol was 100% for the >100 μm HDPE micro-beads.

To assess procedural contamination, three blanks comprising 6 mL RO water were processed in accordance with the sequential extraction protocol. After oven-drying, filters were visually inspected under a dissecting microscope and any particle or fibre present on the filters was treated as procedural contamination. Procedural contamination blanks revealed the presence of hair-like fibres and very fine dark particles <100 μm on each filter as previously reported by Foekema et al. [22]. Because these particles were smaller than 100 μm, they didn’t interfere with the extraction of microplastics sized >100 μm conducted with this protocol. If this protocol is being adapted to investigate microplastics <100 μm, greater care must be taken to prevent procedural contamination. This could include wearing synthetic-free natural fibre clothing [31] or working in a sealed damp-wiped room [32].

#### Protocol suitability

Seven target polymers were used to test the suitability of the sequential extraction protocol: HDPE, polyethylene (PE), poly(ethylene terephthalate) (PET), nylon (polyamide, PA), vinyl chloride/vinyl acetate/vinyl terpolymer (PVC), (aminoethyl) polystyrene (AM-PS) and polypropylene (PP). Ten pieces (1 cm long, cut using a scalpel) and 20 particles (< 1 mm^2^, grated) were prepared from each polymer type. These pieces and particles were then treated with acid digestion (see *Acid digestion of organic materials*) and emulsification (see *Emulsification of fats*), sequentially. Four tests were run to assess potential physical and chemical degradation:

1 The area of each of the ten pieces (1 cm long) per polymer type was measured using the software Image J^®^ before and after acid digestion followed by emulsification. A Student's *t*-test (two-tailed, paired samples) was run with a null hypothesis of no change in the area of the pieces before and after the digestion and emulsification methods.
2 Twenty particles (<1 mm^2^) per polymer type were also subjected to acid digestion followed by emulsification. Particles were then manually sorted and visually counted.
3 Ten pieces (1 cm long) of the target polymers that were recovered after acid digestion and emulsification (i.e. not completely degraded) were subjected to density separation (NaCl, 1.2 g/cm^3^). The capacity of each treated polymer to float or sink was recorded.
4 ATR-FTIR spectroscopy was used to determine whether the sequential extraction protocol affected polymer identification. The polymer composition of each plastic piece was measured prior to treatment using FTIR spectroscopy. Pieces were then treated with the acid digestion followed by emulsification. Pieces that were recovered after these two methods were rinsed with RO water and oven-dried at 60 °C for 4 h before repeating the chemical analysis using FTIR spectroscopy. A percent similarity, calculated by comparing the FTIR spectra of the treated polymers against those of the untreated polymers (PerkinElmer COMPARE algorithm), was used to assess chemical degradation.

HNO_3_ digestion completely dissolved all pieces and all particles of PET and PA. The protocol did not affect PE, HDPE, PVC, AM-PS and PP; 100% recovery was achieved and no change in size or change in polymer identification was observed after exposure of these five polymer types to acid digestion and emulsification.

The density separation method is suitable for polymers with a density lower than the hypersaline brine, i.e. ρ < 1.2 g/cm^3^. Of all polymers inert to HNO_3_ digestion (i.e. PE, HDPE, PVC, AM-PS and PP), only PVC was not recovered by density separation, which was expected as its density is higher than the hypersaline brine (PVC ρ = 1.16–1.58 g/cm^3^, hypersaline NaCl ρ = 1.2 g/cm^3^). To recover a wider range of high-density polymers including PVC, solutions with a density >1.2 g/cm^3^, such as sodium polytungstate (1.4 g/cm^3^ [33]), or sodium iodide (1.6 g/m^3^ [31]) could be used.

A limitation of using HNO_3_ digestion as a separation method is a notable discolouration of the target polymers tested. This makes comparison with other studies difficult, as most studies on microplastic ingestion by marine biota rely on visual inspection using microscopy as the primary identification technique [1]. For this reason, we recommend using FTIR spectroscopy [19,30,34], which allows comparable results on polymer identification and is gaining traction as it becomes less expensive and more sensitive.

### Results

This sequential extraction protocol allows the extraction of microplastics from green turtle chyme with 100% efficiency for >100 μm microplastics of five common plastic pollutants: PE, HDPE, AM-PS, PP and PVC (PVC is only extractable if density separation is not used, i.e. if samples don’t comprise sediment). Four of the polymer types deemed suitable for this extraction protocol represent 70% of the plastics produced globally in 2007: HDPE = 21%, PP = 24%, PS = 6%, PVC = 19% [35].

Using this sequential extraction protocol, two macroplastics and seven microplastics; two paint chips and five mixed-yarn synthetic plastic particles, were found in the foregut of two green turtles from Australia’s Great Barrier Reef [36]. These results highlight the need for analysis of an increased sample size in order to improve our knowledge of the microplastic load of sea turtles from the Great Barrier Reef World Heritage Area and around the world. The sequential extraction protocol validated in this study will enable researchers worldwide to quantify green turtles microplastic contamination in a reliable and comparable way.
