# Supplementary material for: Validation of an optimised protocol for quantification of microplastics in heterogenous samples: A case study using green turtle chyme
Source: MethodsX. 2018 Aug 10;5:812–23. doi: 10.1016/j.mex.2018.07.009 (PMC6092311; doi:10.1016/j.mex.2018.07.009)
Supplement: Supplementary file 1 [file mmc1.docx]

**Supplementary material:**

During preliminary trials of the sequential protocol on green turtle chyme, undigested fat residuals were observed after acid digestion. As undigested material could trap microplastics and prevent further analysis, trials on homogenised chyme samples (see *Sample preparation*) were run to determine a method to eliminate fat residuals:

1. Chyme samples were subjected to alkaline digestion

Materials:

- Aluminium foil
- Block heater (80 °C, model AIM500, SEAL Analytical)
- Reverse osmosis (RO) water
- Sodium hydroxide pellets (NaOH, Merck, CAS number 131-73-2)

Procedure:

- 12.60 g of NaOH pellets and 20 mL of RO water were added to chyme samples of 20 g wet weight.
- Samples were covered with aluminium foil and were left overnight at room temperature (~20 °C).
- Samples were uncovered and heated for 10 h at 80 °C in a block heater. Samples were visually inspected every 30 min and digestion was allowed to continue if undigested material remained.

While a portion of sample was digested within 30 min, the rest of it remained undigested after 10 h of heating (Figure 1). As this alkaline digestion was incomplete, it was considered unsuitable for microplastic extraction from green turtle chyme.


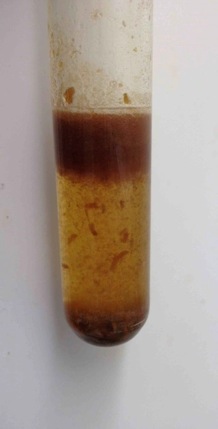


Figure 1: Materials remaining after sodium hydroxide digestion of green turtle chyme: overnight digestion at room temperature (~20 °C) followed by 10 h at 80 °C.

2. HNO_3_ digestion of chyme samples followed by alkaline digestion of remaining materials

Materials:

- Aluminium foil
- Block heater (80 °C, model AIM500, SEAL Analytical)
- Nitric acid (HNO_3_, 69.5%, Scharlau)
- RO water
- Sodium hydroxide pellets (NaOH, Merck, CAS number 131-73-2)

Procedure:

- HNO_3_ digestion of homogenised chyme samples was conducted as described in *Acid digestion of organic materials*.
- Remaining materials were subjected to an overnight alkaline (NaOH) digestion (described above).

After residuals of the acid digestion were re-suspended in the NaOH solution and left overnight, no further digestion was observed (Figure 2). As the alkaline digestion was incomplete, this acid:alkali digestion method was considered unsuitable for microplastic extraction from green turtle chyme. These results contrast with Roch and Brinker (2017), who successfully extracted microplastics from gastrointestinal tract of fishes using NAOH and HNO_3_ digestions sequentially. This difference highlights the need of an adapted extraction protocol for species with different diets.


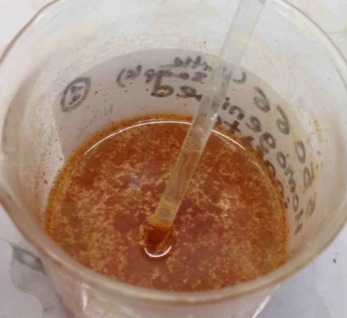


Figure 2: Undigested materials remaining after acid:alkali digestion of green turtle chyme.

3. Finally, a re-suspension of remaining materials in a warm sodium lauryl sulphate solution (described in *Emulsification of fats*) was trialled. As all fat residues were successfully emulsified, this method was considered suitable for microplastic extraction from green turtle chyme samples, and was incorporated into the sequential extraction protocol.

**References**

S. Roch and A. Brinker, Rapid and efficient method for the detection of microplastic in the gastrointestinal tract of fishes, *Environ. Sci. Technol*. **51**, 2017, 4522–4530, http://doi.org/10.1021/acs.est.7b00364.
